# Current trends in art and design for paediatric wellness in built environments (2020–2025): a thematic review

**DOI:** 10.3389/fped.2025.1666934

**Published:** 2026-01-12

**Authors:** Mohd Zairul, Noor Hafizah

**Affiliations:** 1 Department of Architectural Engineering, College of Engineering & Advanced Computing, Alfaisal University, Riyadh, Saudi Arabia; 2 Faculty of Medicine & Health Sciences, Universiti Putra Malaysia, Serdang, Selangor, Malaysia

**Keywords:** art & design, built environment, sustainable design, thematic review, wellness, pediatric wellness

## Abstract

Art and design interventions are increasingly recognized as necessary for paediatric wellness in built environments (e.g., hospitals and schools). However, current literature is fragmented, lacking a holistic integration of design strategies to address children's psychological, sensory, and physical needs. This review addresses that gap by systematically examining recent studies linking design strategies to paediatric well-being. We aimed to identify prevailing trends and themes in publications from 2020 to 2025. Using a systematic thematic review approach (TreZ), we searched Scopus, Web of Science, and Google Scholar, identifying 25 relevant studies after applying strict inclusion criteria. Five major thematic domains emerged: (1) biophilic and nature-based design, (2) playful and interactive environments, (3) sensory and emotional design, (4) inclusive and family-centred design, and (5) aesthetic, cultural, and identity-oriented design. The findings reveal a lack of interdisciplinary integration and limited empirical validation of current design approaches. This review underscores the need for cross-thematic integration, greater child participation in design, rigorous post-occupancy evaluation, and exploration of smart technologies to create more restorative, health-supportive environments for children.

## 1 Introduction

The integration of art and design into paediatric healthcare environments has been shown to significantly enhance the well-being of children and their families. Incorporating child-friendly aesthetics, such as colourful and visually appealing spaces, is particularly beneficial in healthcare settings as it helps distract young patients from the stressors associated with illness and treatment. Mutambo et al. (1) emphasised that children are often more engaged in healthcare settings when the environment is dynamic and colourful, leading them to temporarily forget their purpose for being there (1). Similarly, research in Malaysia indicates that bright colours create a cheerful ambiance in paediatric wards, underscoring the importance of visual stimuli in such settings (2). Moreover, the concept of “positive distractions” a term encompassing various forms of art, play, and environmental aesthetics, has been highlighted as a crucial factor in enhancing children's emotional well-being and healthcare experiences (3).

Such distractions not only help reduce anxiety and stress during medical procedures but also improve overall satisfaction with healthcare experiences (3). Environments constructed with specific spatial arrangements and opportunities to socialise further support children's emotional and psychological health, demonstrating that art and environmental aesthetics are not merely decorative but also functionally therapeutic (3). Incorporating nature and green spaces into healthcare environments can also provide significant benefits. Russo and Andreucci discussed how biophilic design, an approach that connects architecture with nature, can promote children's physical activity and mental health, suggesting that access to green environments directly influences health outcomes (4). Khatavkar (5) emphasised the importance of outdoor activities and green space accessibility in urban environments to maintain children's physical and mental health, suggesting that well-designed public spaces can foster better health outcomes (5). This is echoed in research showing that access to quality neighbourhoods significantly correlates with improved mental health outcomes for children (6).

The amalgamation of the art, nature, and purposeful design of these elements can be framed within the broader perspective of creating supportive environments, not only in healthcare facilities but also in urban planning and community design. Alderton et al. affirmed that neighbourhood-level interventions are vital to fostering child mental health, insisting that design elements such as public open spaces are crucial for achieving such outcomes (6). Likewise, Jansson et al. noted that child-friendly initiatives, such as those advocated by UNICEF, aim to incorporate the perspectives of children when environments are being designed for them, indicating a paradigm shift towards more inclusive urban design strategies (7). Ultimately, the role of art in healthcare environments is not simply a superficial addition but one that requires thoughtful integration that resonates with children's unique experiences of hospitalisation (8).

This situation demands a comprehensive understanding of children's needs and preferences during the design process of healthcare environments. In conclusion, the intersection of art, design, and nature in paediatric healthcare settings creates multifaceted environments that can significantly enhance children's emotional and psychological well-being. These environments are pivotal for not only immediate healthcare experiences but also fostering improved long-term health outcomes. This paper aimed to conduct a thematic review of all relevant articles sourced from reputable databases, including SCOPUS, WoS, and Google Scholar, to address the following research question:

RQ: What are the current trends and patterns in art and design for the wellness of paediatrics in the built environment from 2020 to 2025?

## 2 Methodology

The term “thematic review” (TR), or TreZ, using ATLAS.ti 25 as the tool introduced by Mohd Zairul (9); Hafizah et al. (10); Mnea and Zairul (11, 12); Zairul (13), is applicable because the method followed in this study applied a thematic analysis procedure in the literature review. The TreZ method is a type of literature review that involves identifying, analysing, and synthesising themes or patterns within the research on a particular topic. Using software like ATLAS.ti can be helpful in this process because it enables more efficient and accurate analysis of the data, and it can help identify patterns and themes within the research. In a TR using ATLAS.ti and Mendeley, the software can be used to manage and reference literature, define research topics and questions, represent theoretical frameworks, incorporate research materials, define factual features, and develop patterns and areas of analytic interest. The software allows users to create coding schemes, annotate texts, and identify patterns and themes within the data.

Using software like ATLAS.ti can benefit the review process because it enables more efficient and accurate analysis of the data compared to a conventional approach like SLR or Scoping review. It can also help ensure consistency and rigour in the analysis by providing a systematic approach to coding and theme identification. However, it is important to note that using software like ATLAS.ti requires time and effort to be invested in learning how to use the tool effectively. It is also important to maintain flexibility and openness in the analysis process while remaining systematic and consistent in the approach. It is not uncommon for qualitative data analysis software like ATLAS.ti to be used in the process of conducting a review paper, including the TR approach. While it may not be used in every review paper, it can be a valuable tool for organising and analysing large amounts of qualitative data, such as text from research articles or interview transcripts. Unlike other approaches, such as systematic literature reviews (SLRs), scoping reviews, and meta-analyses, may also be used, depending on the specific aims and objectives of a review. Clarke and Braun (14) defined thematic analysis as a process of identifying patterns and constructing themes by way of a thorough reading of the subject.

### 2.1 TreZ (thematic review flowZ)

**Figure 1:**
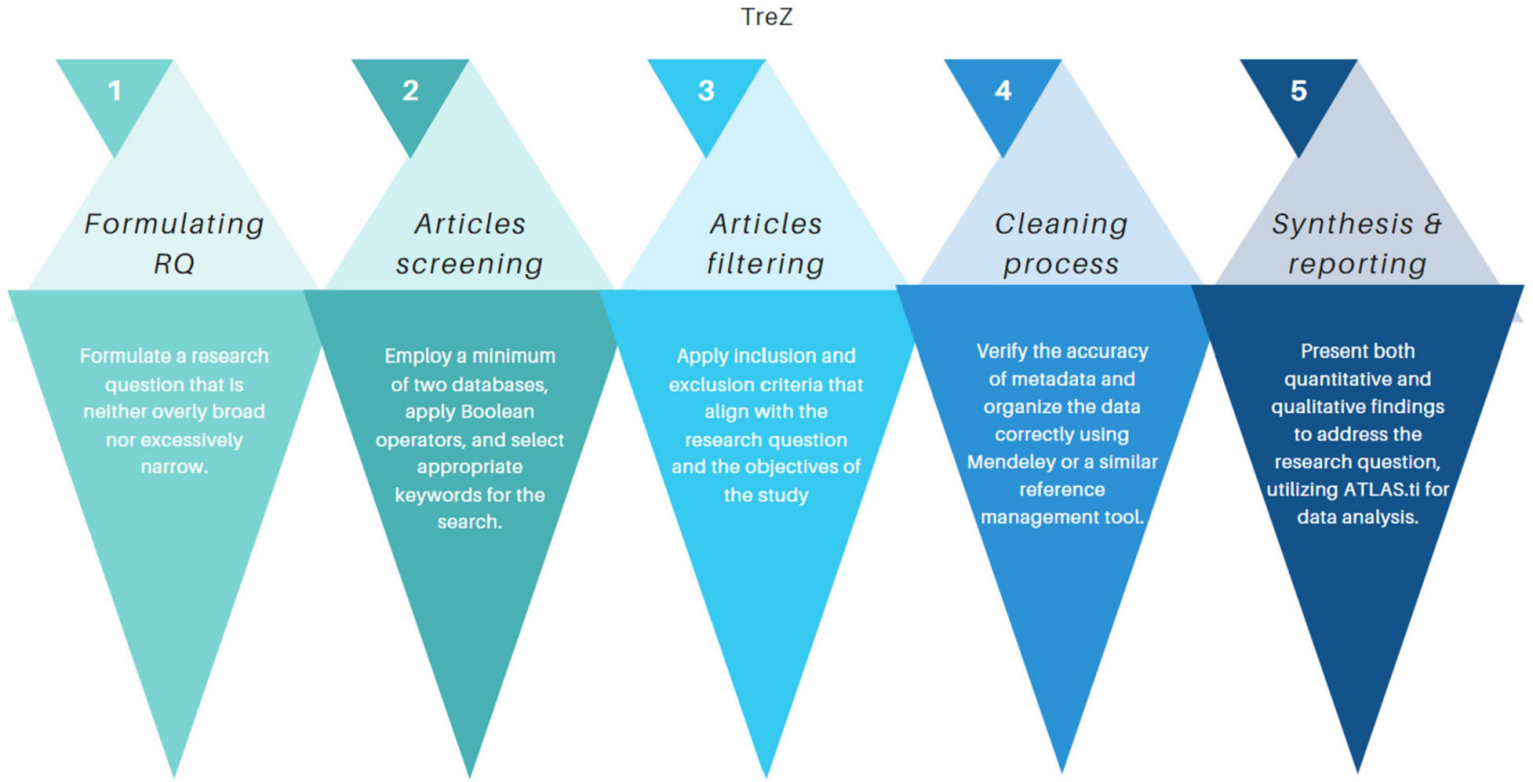
Thematic review flowz (TreZ).

The **TreZ Thematic Review FlowZ**, developed by Zairul (15), provides a systematic and rigorous approach for conducting thematic reviews in research. The framework consists of five interconnected steps, each designed to ensure that the review process is transparent, replicable, and aligned with the research question (Figure 1).

- Step 1: Formulating the Research Question (RQ)

The first step emphasizes the importance of framing a research question that is neither too broad nor too narrow. A well-defined RQ serves as the foundation of the review, ensuring clarity in scope and direction. By striking the right balance, the review avoids unnecessary complexity while maintaining sufficient depth to capture meaningful insights.

- Step 2: Articles Screening

In the second step, the process moves to systematic searching across a minimum of two academic databases. Boolean operators are employed alongside carefully selected keywords to ensure comprehensive retrieval of relevant literature. This step is critical for capturing a broad yet focused dataset that reflects the state of knowledge in the chosen area.

- Step 3: Articles Filtering

Following the initial screening, inclusion and exclusion criteria are applied to refine the dataset. These criteria are tailored to align with both the research question and the objectives of the study. This ensures that only relevant, high-quality articles are retained, reducing noise and improving the reliability of subsequent analysis.

- Step 4: Cleaning Process

The fourth step addresses data accuracy and organization. Metadata is verified for correctness, and references are systematically managed using tools such as Mendeley or similar platforms. This process safeguards against errors, eliminates duplicates, and ensures that the dataset is structured in a way that facilitates rigorous analysis.

- Step 5: Synthesis and Reporting

The final step involves synthesizing the filtered and cleaned literature into coherent themes and insights. Both quantitative and qualitative findings are presented, with data analysis supported by tools such as ATLAS.ti. This dual emphasis allows the review to address the research question comprehensively, capturing not only statistical patterns but also nuanced qualitative perspectives.

### 2.2 Search strategy

This study adopted the TreZ approach to thematic reviews, employing a triangulated search across three major databases: Scopus, Web of Science (WoS), and Google Scholar. Each database was selected for its unique strengths and coverage. In Scopus, the search string combined the terms *paediatric*, *built environment*, and *art and design*, with the additional keyword *health* to ensure comprehensive coverage. Results were limited to peer-reviewed journal articles written in English, published in final form, and available as open access. This search yielded 91 records. In WoS, all fields (AF) were targeted using the string: *Art and Design OR therapeutic* AND *paediatrics OR children* AND *built environment OR architecture*. Refinements excluded review articles, early access publications, conference proceedings, editorials, meeting abstracts, book chapters, and retracted publications. Only English-language, open-access journal articles were retained. Within the 2020–2025 timeframe, the search produced 118 records. In Google Scholar, a similar keyword combination was applied: *wellness*, *paediatric*, *built environment*, and *art and design*. The search was restricted to the years 2020–2025, resulting in 128 records. While Google Scholar provided a wider range of document types, including grey literature, this breadth ensured the inclusion of emerging ideas not consistently indexed in Scopus or WoS (Table 1).

**Table 1:** Search strings from scopus, WoS, and google scholar.

Indexed	Search strings	Results
SCOPUS	(TITLE-ABS-KEY (paediatric) AND TITLE-ABS-KEY (“built environment”) AND TITLE-ABS-KEY (“Art and design”) OR TITLE-ABS-KEY (health)) AND (LIMIT-TO (DOCTYPE, “ar”)) AND (LIMIT-TO (SRCTYPE, “j”)) AND (LIMIT-TO (LANGUAGE, “English”)) AND (LIMIT-TO (PUBSTAGE, “final”)) AND (LIMIT-TO (OA, “all”))	91 results
WoS	Art and Design OR therapeutic (All Fields) AND paediatrics OR children (All Fields) AND built environment OR architecture (All Fields) and Review Article or Early Access (Exclude—Document Types) and Proceeding Paper or Editorial Material or Meeting Abstract or Book Chapters (Exclude—Document Types) and Turkish or German or Spanish or Hungarian (Exclude—Languages) and Retracted Publication (Exclude—Document Types) and All Open Access (Open Access) [Year 2020–2025]	118 results
Google scholar	Wellness AND paediatric AND “Built Environment” AND “Art and Design” AND (Year: 2020–2025)	128 results

### 2.3 Eligibility criteria

To maintain the quality and relevance of the dataset, strict **inclusion and exclusion criteria** were applied.

1. **Inclusion criteria:** Full-length empirical papers, published in English, and directly addressing the intersection of *children*, *wellness*, and the *built environment*.
2. **Exclusion criteria:** Review papers, secondary analyses such as policy reviews, conceptual-only papers, conference proceedings, editorials, book chapters, meeting abstracts, retracted publications, and studies focusing primarily on adolescents or other non-paediatric populations.

### 2.4 Screening and filtering

**Figure 2:**
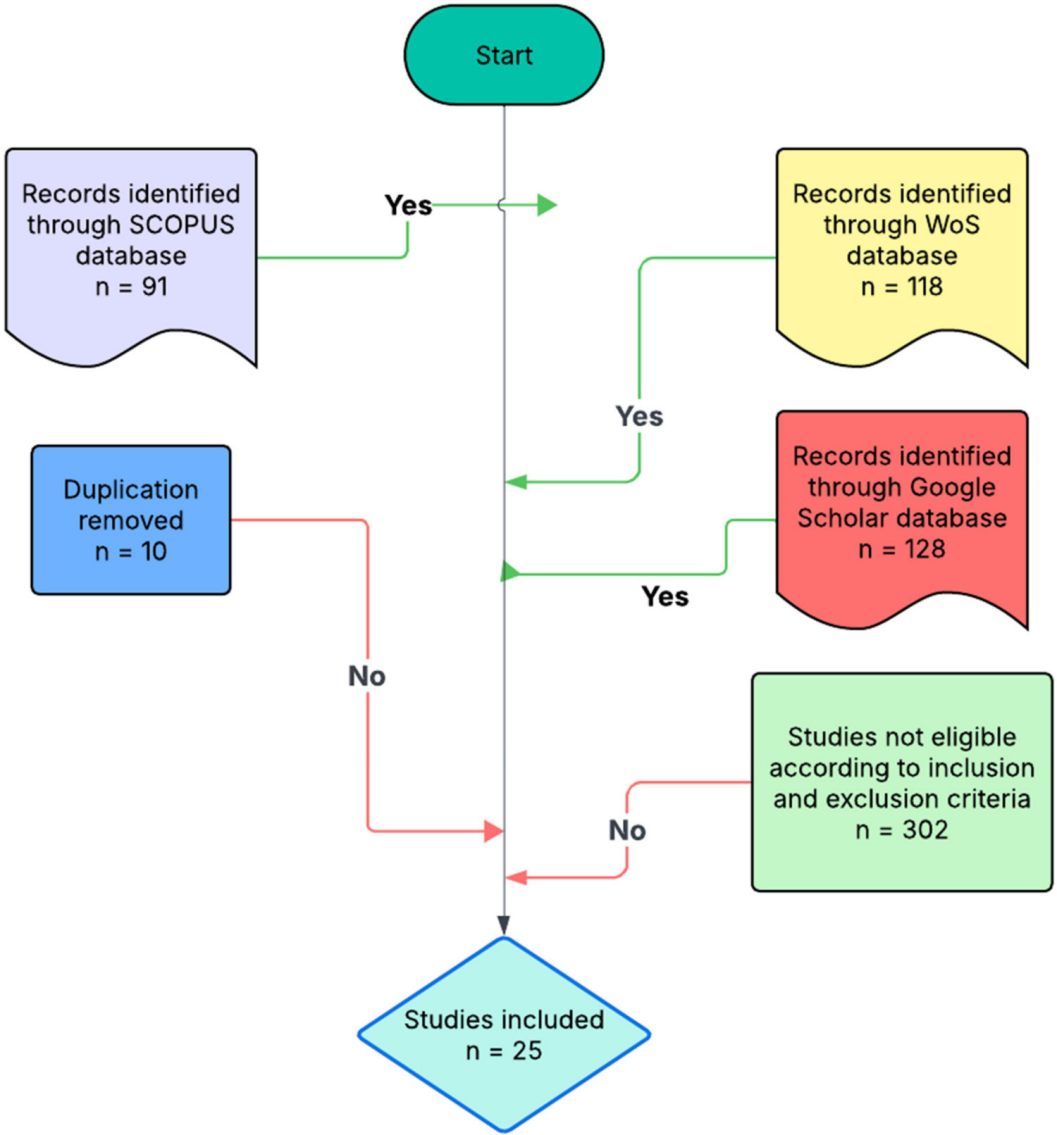
Inclusion and exclusion criteria (TreZ method).

The initial combined search across the three databases produced 337 records. After duplicate removal (10 records), the remaining articles were screened using the eligibility criteria. In total, 302 records were excluded, leaving a final dataset of 25 empirical studies. The triangulated use of Scopus, WoS, and Google Scholar ensured methodological robustness and comprehensive coverage. Scopus contributed high-quality, peer-reviewed literature; WoS enhanced reliability through targeted topic filtering and extended temporal scope; and Google Scholar complemented these with broader coverage, capturing contemporary trends and emerging discussions. The final dataset represents a rigorously selected evidence base for examining trends in art and design for paediatric wellness within the built environment (Figure 2).

## 3 Results

This section presents systematically the findings of the thematic review; it is structured into two distinct parts: quantitative and qualitative analyses. The quantitative analysis provides descriptive insights into the characteristics of the reviewed studies, employing visual aids such as a Sankey diagram and summary tables to clearly illustrate data distributions, publication trends, and intervention types. The qualitative analysis subsequently presents a detailed narrative examination of the identified themes, interpreting the core insights on how art and design interventions influence paediatric wellness within built environments.

### 3.1 Quantitative results

#### 3.1.1 Sankey diagram

**Figure 3:**
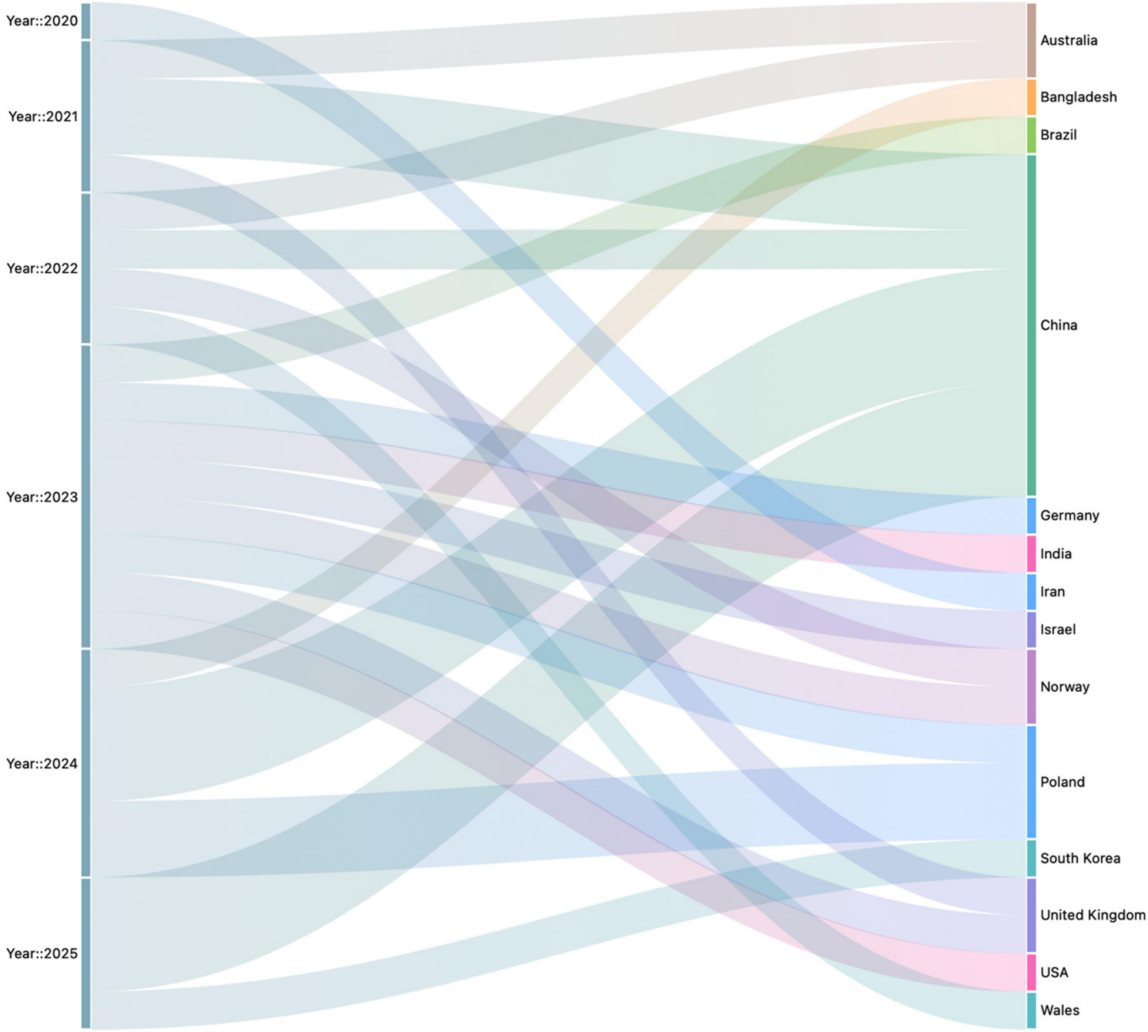
Sankey diagram for year vs. country.

The distribution of the 25 selected studies across countries and years reveals both temporal growth and geographical diversity. In terms of yearly trends, research activity began modestly in 2020 with a single study, followed by four studies each in 2021 and 2022. A notable increase occurred in 2023 with eight publications, while 2024 recorded six studies, and 2025 produced four. Geographically, China contributed the largest share with nine publications, spanning from 2021 through 2025, including a peak of three studies each in 2024 and 2025. Poland followed with three studies distributed across 2022 and 2024. Australia, the United Kingdom, and the United States each contributed two studies across multiple years, while Norway also accounted for two studies, one in 2022 and another in 2023 (Figure 3).

**Figure 4:**
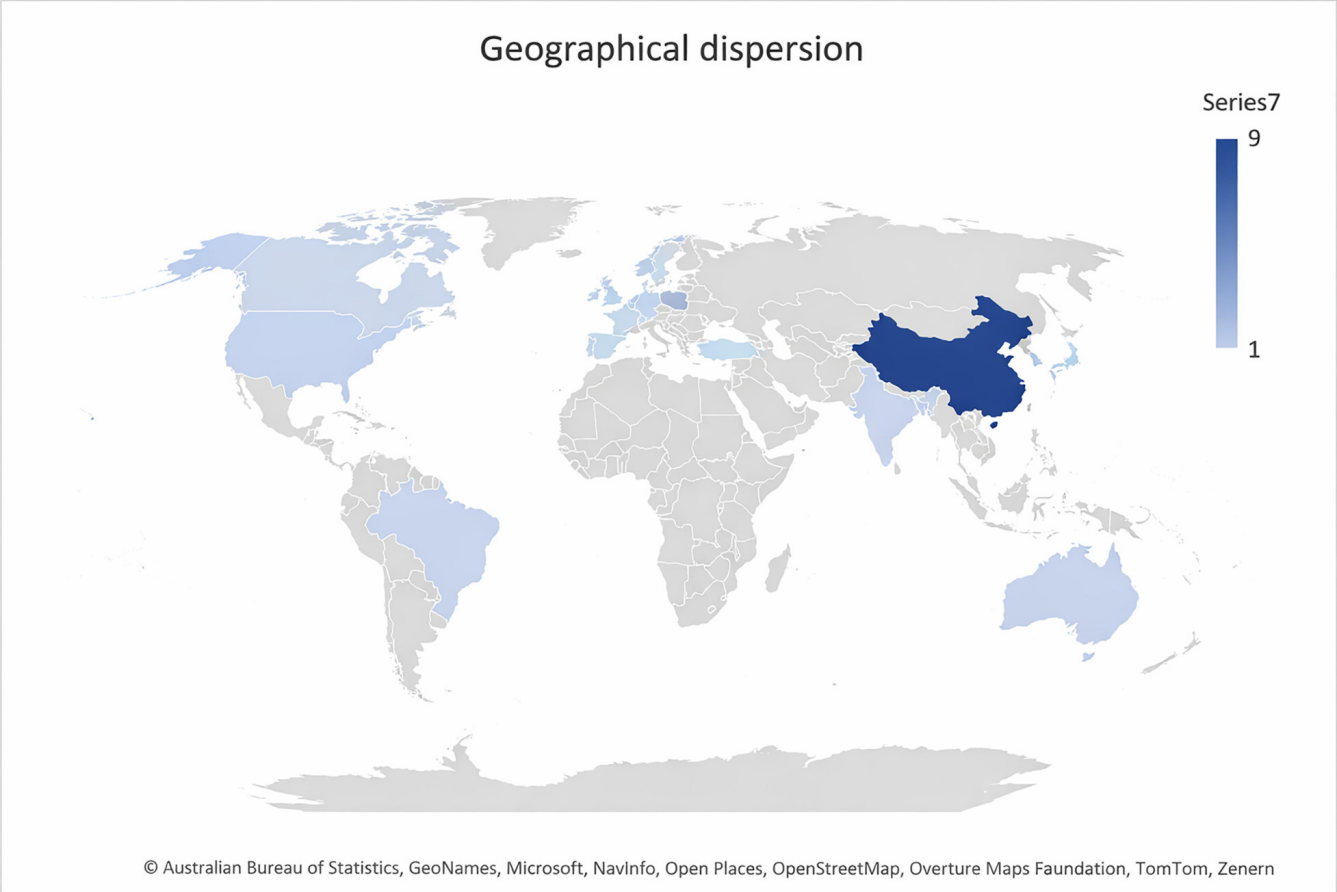
Geographical dispersion.

The remaining countries, Bangladesh, Brazil, Germany, India, Iran, Israel, South Korea, and Wales, each contributed one study within the period. Overall, the data indicate a growing global engagement with paediatric wellness and built environment research, with China emerging as the most consistent and dominant contributor, complemented by steady but smaller contributions from Europe, North America, and parts of Asia (Figure 4).

#### 3.1.2 Geographical dispersion

#### 3.1.3 Journal distribution

The distribution of the 25 selected studies across journals demonstrates both thematic diversity and concentration within specific outlets. The journal Buildings accounted for the largest share with five publications, predominantly in 2024 (*n* = 3) and 2025 (*n* = 2), highlighting its role as a central platform for research on paediatric wellness and the built environment. Sustainability followed with two contributions spread across 2024 and 2025, while the International Journal of Environmental Research and Public Health published two studies in 2021 and 2022. Similarly, the Journal of Asian Architecture and Building Engineering contributed two articles, both in 2023. in specialized journals such as Buildings and Sustainability, suggesting growing recognition of the topic within architecture, design, and sustainability research communities (Table 2).

**Table 2:** Journal distribution based on year of publication.

Journal name	Year of publication					Totals
	2021	2022	2023	2024	2025	
Ambiente Construído	—	—	1	—	—	**1**
Arts	—	—	1	—	—	**1**
BMJ Open	—	1	—	—	—	**1**
Buildings	—	—	—	3	2	**5**
Continuity in Education	—	—	1	—	—	**1**
ECOPSYCHOLOGY	—	—	1	—	—	**1**
Environmental Research	—	—	1	—	—	**1**
European Journal of Sustainable Development	—	—	1	—	—	**1**
Health Environments Research and Design Journal	—	—	1	—	—	**1**
Health Science Reports	—	—	1	—	—	**1**
International Journal of Art and Design Education	1	—	—	—	—	**1**
International Journal of Environmental Research and Public Health	1	1	—	—	—	**2**
Journal of Asian Architecture and Building Engineering	—	—	1	—	—	**1**
Journal of Landscape Architecture	—	—	—	1	—	**1**
Obesity Reviews	1	—	—	—	—	**1**
Sensors	—	—	—	—	1	**1**
Sport, Education and Society	1	—	—	—	—	**1**
Sports	1	—	—	—	—	**1**
Sustainability	—	—	—	1	1	**2**
Totals	5	2	9	5	4	**25**

Total publications refer to the number of articles published in the designated journals.

The remaining journals, including *Ambiente Construído*, Arts, BMJ Open, Continuity in Education, Ecopsychology, Environmental Research, European Journal of Sustainable Development, Health Environments Research and Design Journal, Health Science Reports, International Journal of Art and Design Education, Journal of Landscape Architecture, Obesity Reviews, Sensors, Sport, Education and Society, and Sports, each accounted for a single publication. Temporally, publication activity was highest in 2023 with nine studies, followed by 2021 with five, 2024 with five, 2025 with four, and 2022 with two. Collectively, these findings indicate that while research on *paediatric* wellness in the built environment is dispersed across a variety of disciplinary outlets, a concentration is emerging (Table 2).

#### 3.1.4 Distribution of theme based on author

Following the application of the TreZ method and the screening process described earlier, a total of 25 articles were included in this review. These articles were subsequently categorized into five thematic domains to identify quantitative trends and patterns in the literature. The distribution of studies across themes revealed clear variations in emphasis, reflecting the diversity of approaches taken to address art and design for paediatric wellness in the built environment between 2020 and 2025. The thematic analysis identified five dominant themes across the selected studies:

- **Theme 1:** Biophilic and Nature-Based Design,
- **Theme 2:** Playful and Interactive Environments,
- **Theme 3**: Sensory and Emotional Design Approaches
- **Theme 4:** Inclusive and Family-Centered Design
- **Theme 5**: Aesthetic, Cultural, and Identity-Oriented Design.

The largest body of work (*n* = 12) was aligned with biophilic and nature-based design, highlighting the centrality of natural features in shaping children's well-being. Articles within this theme consistently demonstrated the therapeutic value of daylight, views to nature, greenery, and outdoor environments across hospitals, schools, and urban contexts. These studies underscored biophilic interventions as low-cost, high-impact strategies for improving paediatric wellness. The second most substantial theme was sensory and emotional design approaches (*n* = 10). This cluster of articles emphasized the design of spaces that respond to children's sensory needs and emotional states, ranging from art therapy interventions and trauma-informed design to the integration of positive distraction features such as murals, acoustic control, and calming colour schemes. The prominence of this theme indicates a growing recognition that emotional regulation and sensory comfort are integral to healing environments for children.

Aesthetic, cultural, and identity-oriented design emerged as the third largest theme (*n* = 9), with studies focusing on participatory art, intergenerational programs, and culturally embedded design practices. These works illustrate how built environments can serve not only functional and therapeutic purposes but also act as platforms for affirming identity, strengthening cultural continuity, and enhancing children's sense of belonging. The theme of playful and interactive environments comprised eight articles, demonstrating the increasing role of play, exploration, and interactivity in paediatric wellness. Research in this category covered therapeutic playground typologies, outdoor schoolyard redesigns, and interactive technologies, collectively affirming that environments that stimulate creativity and active engagement are linked to positive developmental and social outcomes.

Finally, an inclusive and family-centered design was reflected in seven articles. These studies emphasized child- and family-centered principles, autonomy-supportive features, and co-design approaches that integrate the perspectives of children, parents, and other stakeholders. Research in this theme also highlighted the importance of inclusive planning for neurodiverse populations, pointing to the need for environments that support diverse developmental trajectories.

Notably, overlaps were observed across several themes, underscoring the interdisciplinary and interconnected nature of the field. Biophilic and sensory themes showed the strongest convergence, as natural elements such as daylight and greenery were consistently framed as both sensory comforts and therapeutic interventions. Certain studies, including Hen (41); Khan et al. (16), were represented in multiple themes, reflecting their wide-ranging contributions to understanding paediatric wellness in different design contexts. In sum, the quantitative distribution of the literature demonstrates that while biophilic and sensory approaches dominate recent scholarship, there is also a robust and growing body of research emphasizing aesthetics, cultural identity, interactivity, and inclusivity. These results illustrate that contemporary research into paediatric wellness in the built environment is not confined to a single design paradigm but rather reflects an integrative perspective that recognizes the interplay of natural, sensory, cultural, and social dimensions in shaping children's health and well-being (Table 3).

**Table 3:** Author vs. theme.

Name of Author(s)	Themes				
	Theme 1: biophilic and nature-based design	Theme 2: playful and interactive environments	Theme 3: sensory and emotional design approaches	Theme 4: inclusive and family-centered design	Theme 5: aesthetic, cultural, and identity-oriented design
Jia et al. (17)	—	—	—	—	/
Payam et al. (18)	/	—	—	—	—
Norton-Westwood (19)	—	—	/	—	/
Lima et al. (20)	/	—	/	—	—
Zhou et al. (21)	/	—	—	—	—
Wang et al. (22)	/	—	—	—	—
Nevill et al. (23)	—	—	—	—	/
Yoo et al. (24)	—	/	—	/	—
Pedrick-Case et al. (25)	—	—	—	—	/
Burke et al. (26)	/	—	—	—	/
Wilson et al. (27)	—	—	—	—	/
Hauge, Lindheim, Røtting, and Johnsen (28)	/	—	/	—	—
Wu et al. (29)	/	/	—	—	—
Sanderud et al. (30)	—	/	—	—	—
Fan et al. (31)	/	/	—	—	—
Czalczynska-Podolska (32)	—	/	/	—	—
Deng, Sulaiman, et al. (33)	/	—	—	—	—
Babbu and Haque (34)	—	—	—	/	—
Szustakiewicz (35)	—	—	—	/	/
Shimokura et al. (36)	—	—	/	/	—
Deng, Ismail, et al. (37)	/	—	/	—	—
Gawlak and Banaś (38)	/	—	/	—	—
Khan et al. (16)	/	/	—	/	/
Yildirim Balkan et al. (39)	—	—	/	—	/
Sathyanarayanan and Caldas (40)	/	—	—	—	—

### 3.2 Qualitative results

The thematic synthesis was guided by the central research question:

“What are the current trends and patterns in art and design for the wellness of paediatrics in the built environment from 2020 to 2025?”

**Figure 5:**
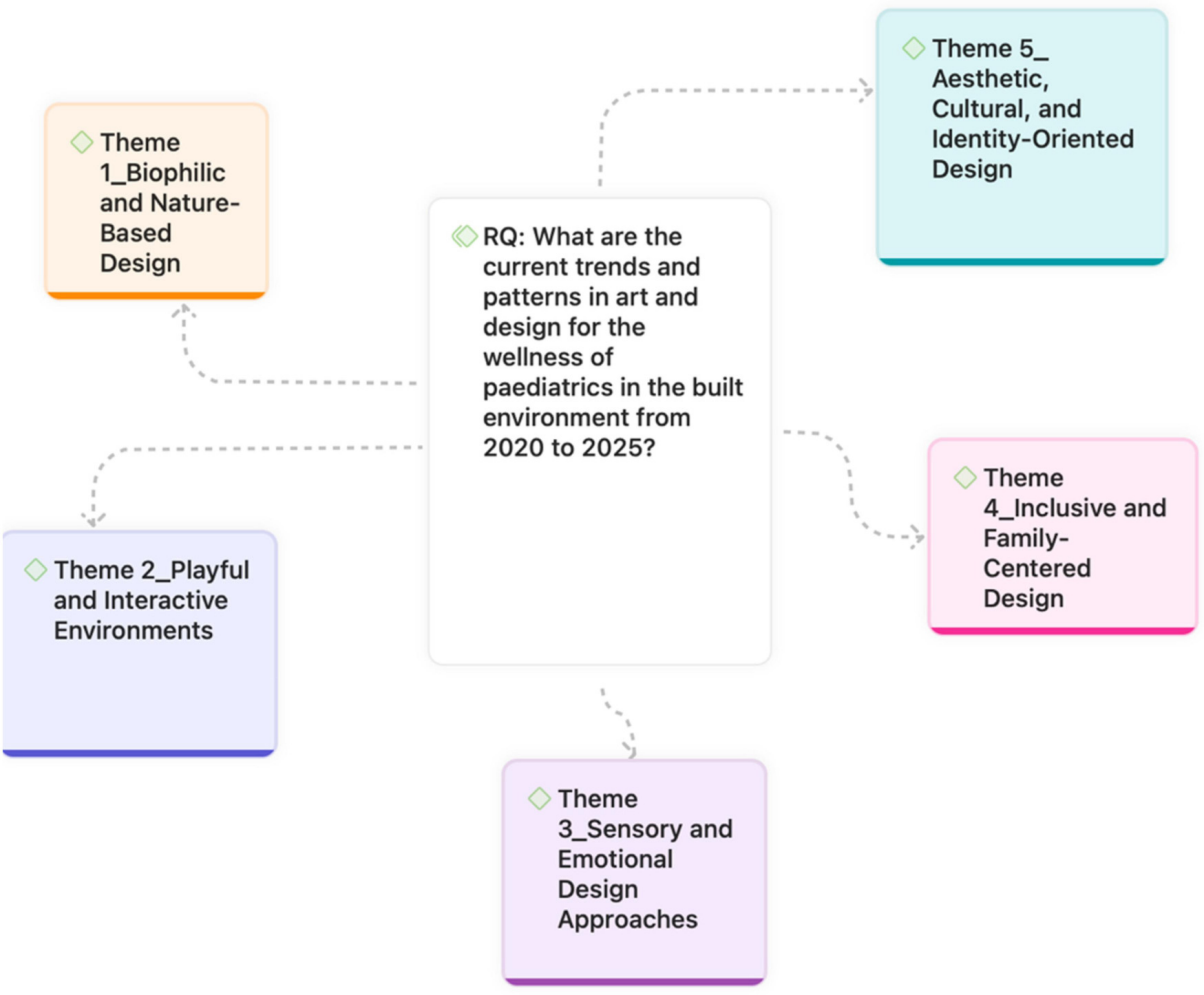
Answering research question.

As illustrated in the figure above, the review identified **five core themes** that reflect the prevailing discourse in the selected literature. These themes collectively represent the multidimensional role of art and design in shaping wellness outcomes for paediatric users in built environments (Figure 5):

1. **Theme 1: Biophilic and Nature-Based Design**

This theme highlights the integration of natural elements, such as daylight, greenery, water, and outdoor spaces, into paediatric environments. Studies demonstrate that these features enhance physical activity, reduce stress, and foster restorative experiences, confirming the therapeutic value of biophilic design across hospitals, schools, and urban settings.

2. **Theme 2: Playful and Interactive Environments**

Emphasizing the role of play, interaction, and exploration, this theme reflects how well-designed play areas, therapeutic playgrounds, and participatory schoolyards can improve children's engagement, learning, and social development. Emerging smart technologies further extend interactivity into the home environment, supporting autonomy and routine.

3. **Theme 3: Sensory and Emotional Design Approaches**

This theme underscores how sensory modulation, trauma-informed design, and positive distraction strategies, such as art therapy, calming colors, murals, and acoustic comfort, help children manage anxiety, regulate emotions, and improve their overall well-being. Neurodiversity-sensitive and age-specific spatial solutions are also central to this theme.

4. **Theme 4: Inclusive and Family-Centered Design**

Focused on social contexts, this theme recognizes the importance of family presence, co-design processes, and inclusivity in shaping paediatric environments. It encompasses both healthcare and educational settings, where participatory design and autonomy-supportive strategies ensure that children and families are active partners in the creation of supportive spaces.

5. **Theme 5: Aesthetic, Cultural, and Identity-Oriented Design**

This theme addresses the cultural and symbolic dimensions of design. It captures how participatory art projects, culturally embedded schoolyard designs, intergenerational art-making, and therapeutic aesthetics contribute to identity formation, belonging, and resilience. These approaches position the built environment as a space not only for healing but also for cultural expression and empowerment.

#### 3.2.1 Theme 1: biophilic and nature-based design

Deng, Ismail, et al. (37) argue that distinct biophilic elements, such as water, plants, and animals, produce unique benefits for children, ranging from resilience to social adaptability. Their claims are reinforced by a similar study by Deng, Sulaiman, et al. (33), who show through intervention studies that children in biophilic-rich kindergartens engage more with nature and develop greater environmental awareness than those in conventional settings. Both studies position early, direct contact with nature as foundational to children's developmental well-being.

**Figure 6:**
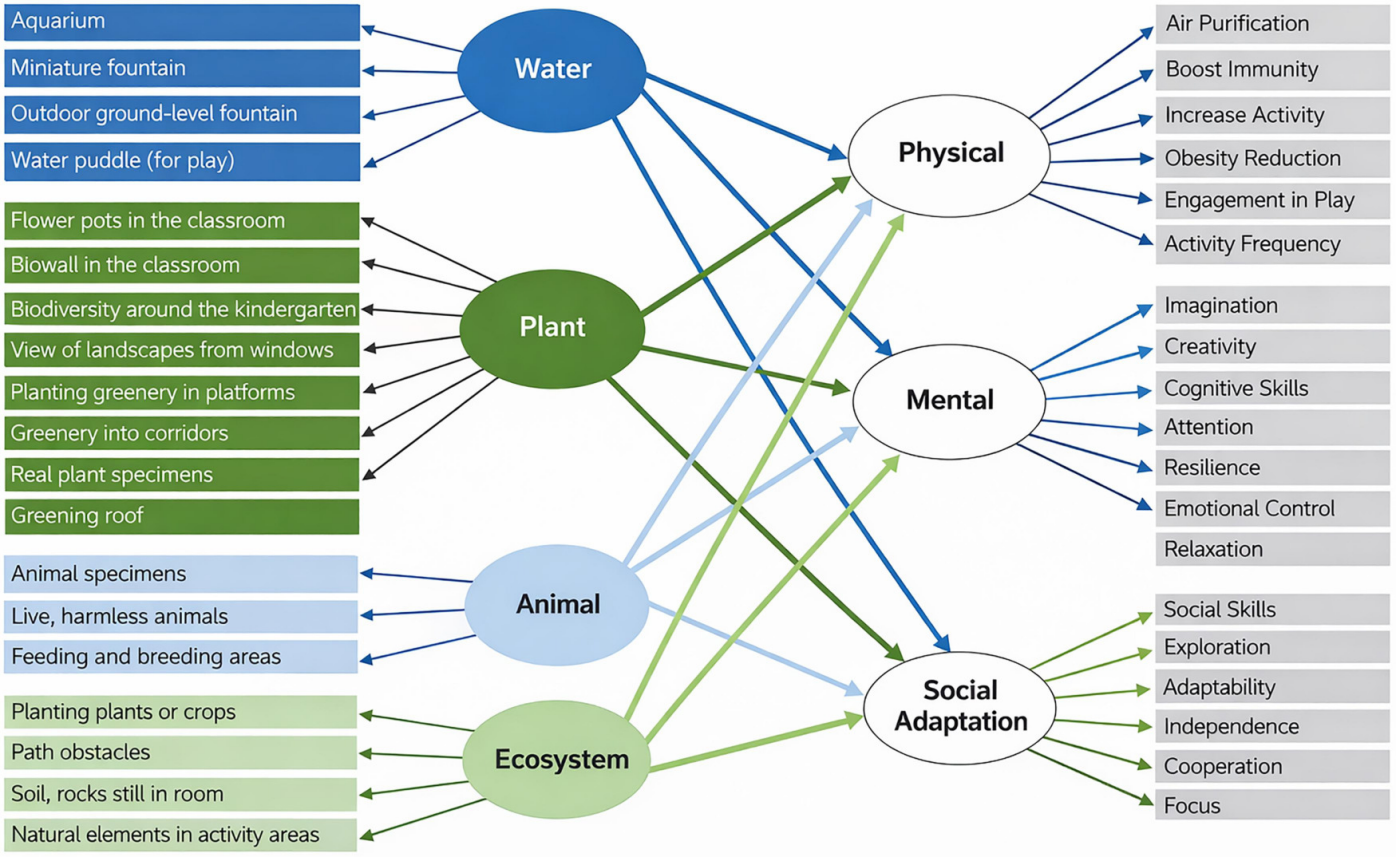
Conceptual framework on biophilic nature by Deng et al. (33, 37).

However, Lima et al. (20) shift the discussion to the clinical context, arguing that even when design incorporates optimal environmental features, such as appropriate lighting, colour schemes, access to natural light and external views, and supportive professional interactions that address physical, psychological, social, and spiritual dimensions of pain, user satisfaction is not guaranteed. Ultimately, they suggest that the decisive factor for many patients and families remains the recovery and cure of the illness, rather than environmental quality alone. Lima et al. (20) challenge Deng et al.'s emphasis on ecological diversity, arguing that even minimal, cost-effective interventions, such as windows, can have a profound impact where natural access is limited (Figure 6). Zhou et al. (21) further nuance this debate by showing that symbolic representations of nature, colorful, playful, and child-like visuals in operating rooms, can ease preoperative anxiety in preschoolers. Their findings suggest that biophilia need not be literal: aesthetic references can substitute for ecological features, particularly in constrained medical environments.

At the urban scale, Wu et al. (29) broaden the argument by linking higher residential greening, lower density, and accessible play facilities to greater play behaviour in children with autism spectrum disorder. This urban-level evidence is expanded by Wang et al. (22), who propose the “Children's 15-Minute Life Circle” framework to ensure equitable access to parks, schools, and clinics. Building from a participatory perspective, Khan et al. (16) show that redesigning schoolyards with native vegetation and playful natural zones improves outdoor learning and activity. These studies collectively extend Deng et al.'s kindergarten focus into wider city and school contexts, positioning biophilic interventions as a low-cost, high-impact strategy across multiple scales.

**Figure 7:**
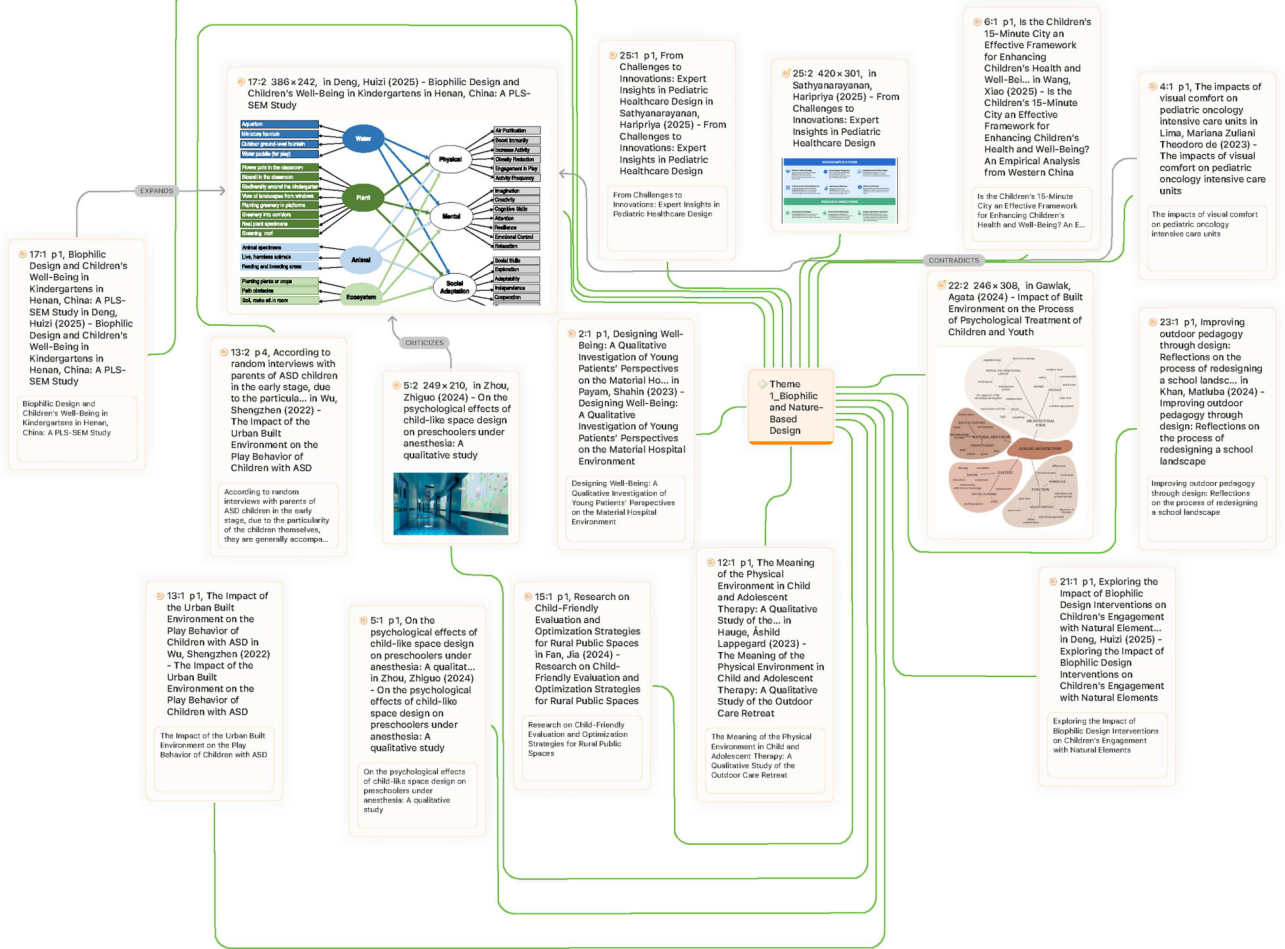
Theme 1 (biophilic and nature-based design).

To summarize, the literature converges on the conclusion that daylight and greenery are universally beneficial, yet diverges on the degree of intervention required. While Deng et al. argue for ecological completeness, Lima et al. and Zhou et al. counter that symbolic or minimal interventions may suffice in clinical contexts. Wu et al., Wang et al., and Khan extend the debate to neighbourhoods and schools, emphasizing systemic and participatory approaches. The unresolved gap lies in determining how these different scales and forms of biophilic design, literal, symbolic, or systemic, interact to deliver consistent health outcomes for children (Figure 7).

#### 3.2.2 Theme 2: playful and interactive environments

This theme highlights designs that encourage play, exploration, and interactive engagement. Play-oriented spaces, whether in schools, playgrounds, or homes, consistently improve children's development and motivation.

**Figure 8:**
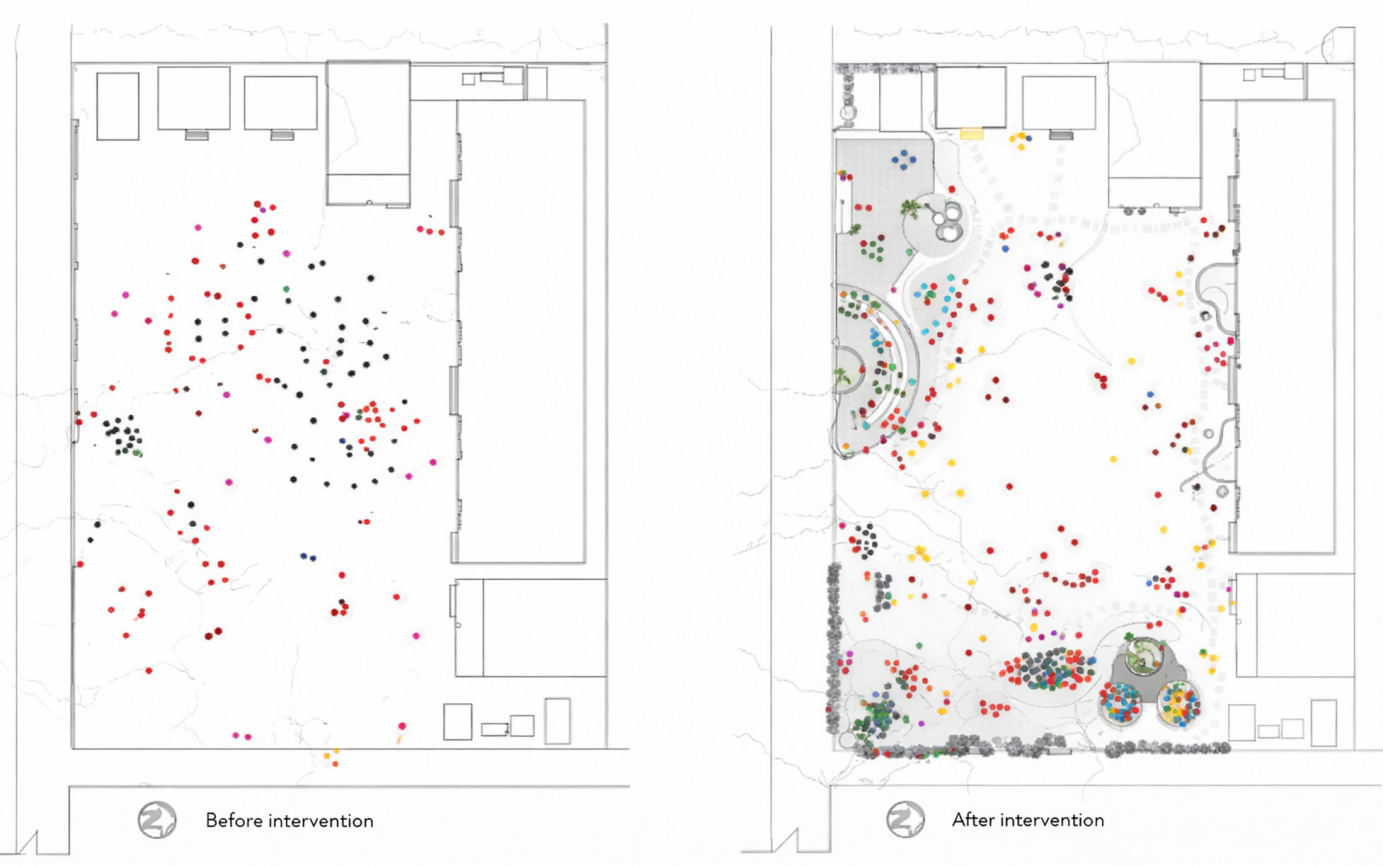
Behaviour maps of the schoolgrounds before and after intervention by Khan et al. (16).

A growing body of scholarship underscores the importance of play-oriented environments for children's well-being. Wu et al. (29) demonstrated that neighborhoods with abundant recreational facilities promote play opportunities for children with autism spectrum disorder (ASD), whereas overcrowded environments significantly constrain participation. Similarly, Sanderud et al. (30) highlighted how teachers in Norwegian kindergartens exercised “didactic sensitivity” by adapting outdoor play settings to children's evolving interests, thereby fostering autonomy. These findings align with Khan et al. (16), work, which showed that participatory redesigns of school landscapes involving both children and teachers increased outdoor engagement and motivation (Figure 8). Collectively, these studies argue persuasively that environments responsive to children's needs promote resilience, autonomy, and participation across diverse settings.

Yet, a counterargument arises when considering the limitations of implementation. Czalczynska-Podolska (32) identified a typology of therapeutic playgrounds that integrate natural, sensory, and thematic elements, but emphasized that most facilities fall short of realizing these ideals in practice. This persistent gap between theoretical best practice and real-world implementation highlights barriers such as budgetary constraints, limited expertise, and competing institutional priorities. Furthermore, while Yoo et al. (24) advanced the notion of ambient sensing technologies in domestic environments to guide children's routines, critics question whether reliance on smart systems risks displacing embodied, social, and imaginative play with technologically mediated routines.

**Figure 9:**
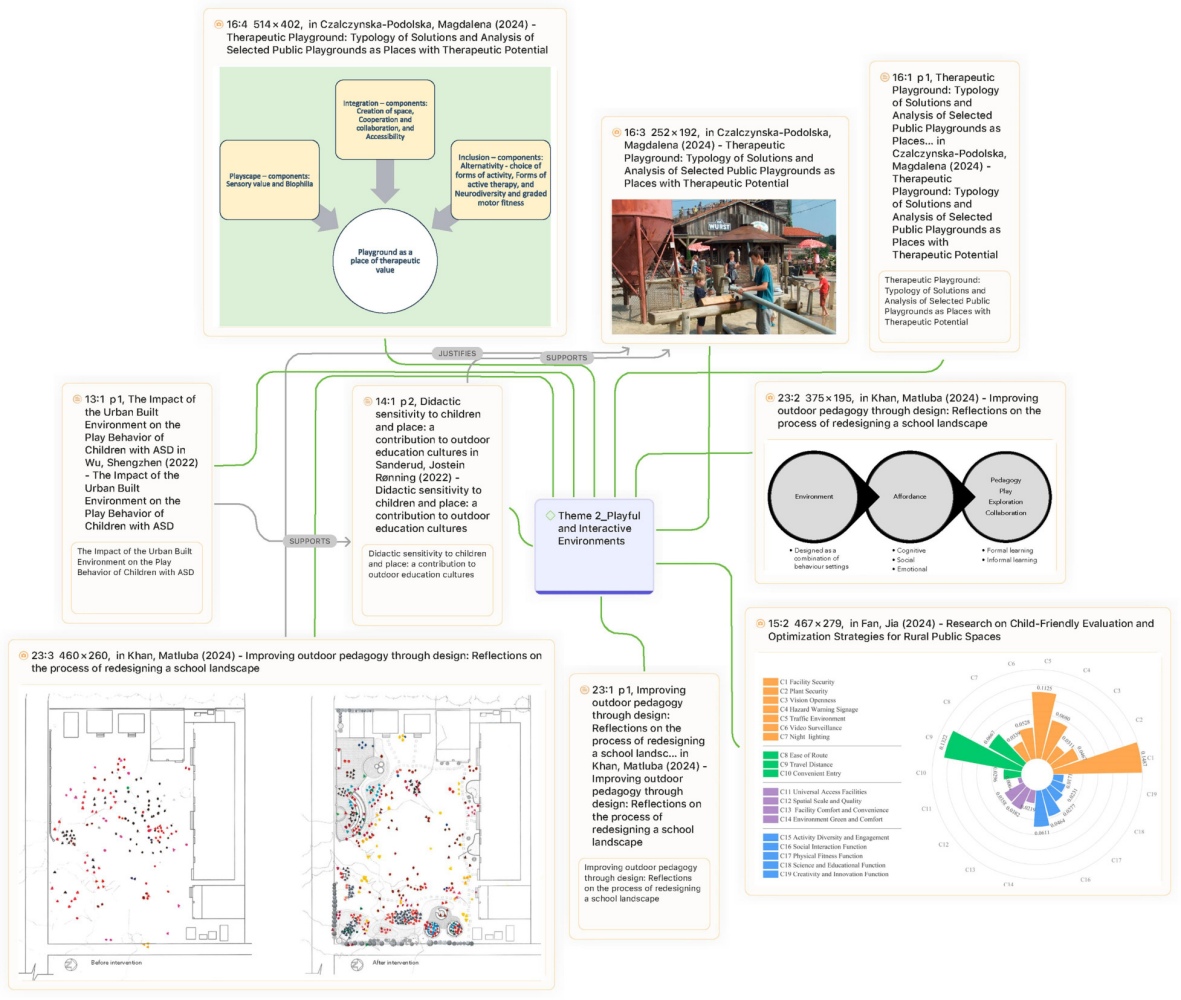
Theme 2 (playful and interactive environments).

The central debate, therefore, is not whether play should be integrated into a designed environment; evidence overwhelmingly affirms its importance, but rather how effectively these ideals can be translated into practice. On one side, proponents argue that participatory and adaptive approaches have demonstrated measurable benefits for children's autonomy and well-being. On the other hand, sceptics caution that under implementation, inequities of access, and an overemphasis on technological mediation may undermine the intended outcomes. Bridging this divide requires not only design innovation but also sustained policy support, investment, and critical reflection on the balance between technological and embodied forms of play (Figure 9).

#### 3.2.3 Theme 3: sensory and emotional design approaches

**Figure 10:**
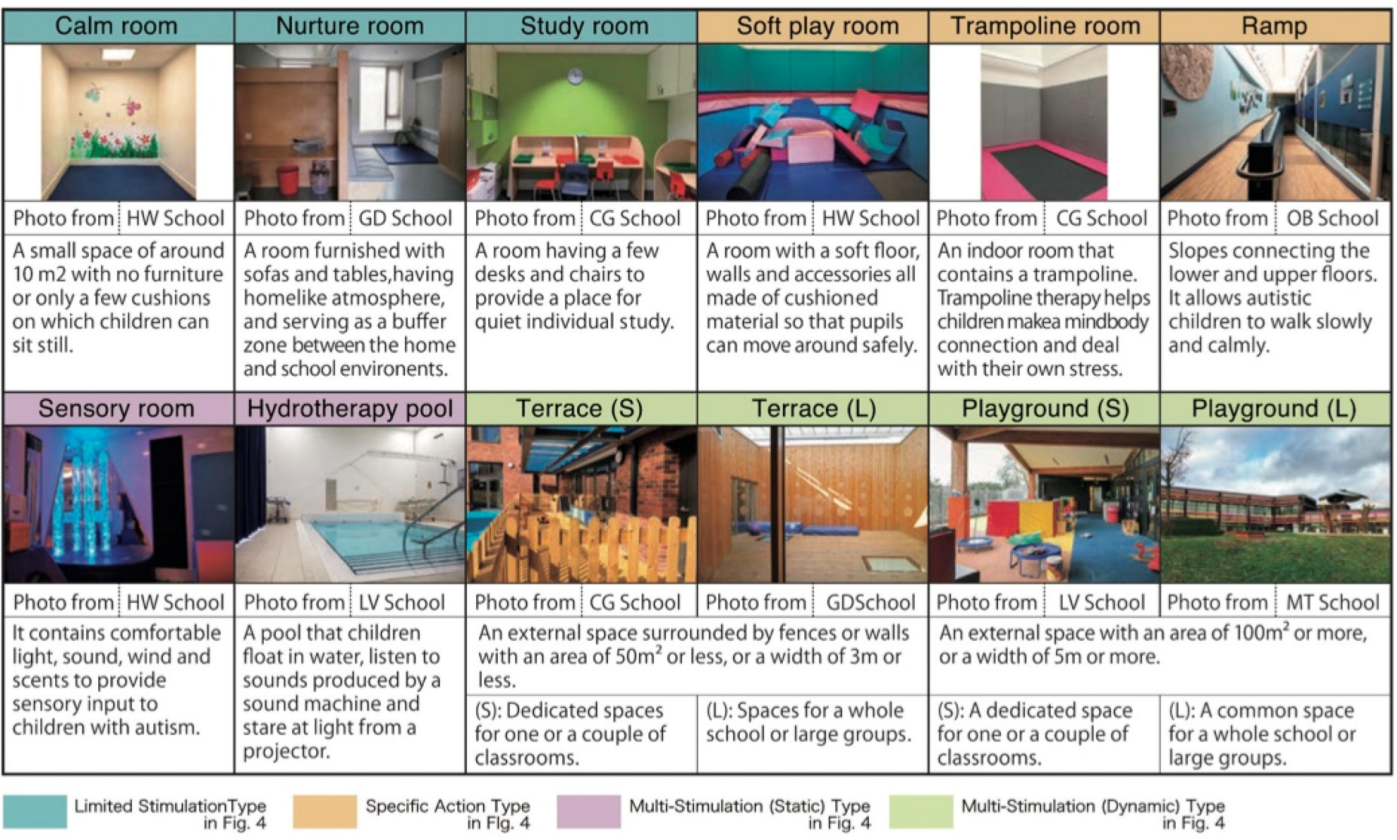
Therapeutic spaces in the schools investigated (36).

This theme foregrounds how sensory and emotional factors, art, light, acoustics, and colour contribute to paediatric well-being, particularly within trauma-informed and neurodiversity-sensitive design approaches. Proponents argue that such design interventions act as powerful non-pharmacological tools to reduce stress and foster emotional regulation. Hen (41) demonstrated that art therapy in hospitals enables children to articulate emotions and cope with disruption, while Yildirim Balkan et al. (39) reported that decorating preoperative wards with familiar cartoons and natural imagery significantly lowered anxiety levels. For neurodiverse populations, Shimokura et al. (36) emphasized age-specific adaptations: calm rooms and outdoor play for younger children vs. mainstream-like settings with sensory accommodations for older children (Figure 10). Similarly, Gawlak & Banaś (38) extended these insights to youth mental health centers, noting that calming aesthetics, participatory design, and user-friendly layouts are crucial to supporting therapeutic alliances. These findings suggest that sensory-rich and emotionally responsive environments can set in motion a “positive circle,” in which natural surroundings and biophilic design, as described by Hauge, Lindheim, Røtting, Johnsen, et al. (28), reinforce trust, emotional safety, and therapeutic engagement.

However, a counterargument highlights that while these interventions show promise, their translation into broader design practice remains inconsistent and fragmented. (Czalczynska-Podolska, 024) Argued that the discourse on therapeutic playgrounds exemplifies this gap: although accessibility concerns have dominated, the more profound issue is integrating therapeutic components, natural, sensory, and social, into public spaces. She identified three pathways for implementing therapeutic playgrounds, but stressed that such approaches are rarely realized comprehensively. Without integrated planning, the potential to transform playgrounds and urban public spaces into inclusive, health-promoting environments remains largely aspirational.

At the clinical level, Lima et al. (20) advanced the debate by framing sensory and emotional design within the broader philosophy of humanized care. Beyond aesthetics, she emphasized the role of adequate lighting, colours, family presence, and psychosocial-spiritual support in sustaining hope and dignity during both recovery and anticipatory mourning. This view cautions against reducing sensory design to superficial add-ons; instead, it demands a holistic integration into healthcare that attends equally to physical, emotional, and existential needs. Taken together, the debate hinges on whether sensory and emotional design is understood as a core therapeutic strategy or as a supplementary enhancement. Proponents underscore its demonstrable benefits in reducing anxiety, fostering autonomy, and strengthening therapeutic relationships. Critics, however, point to systemic barriers—fragmented implementation, limited resources, and the risk of aesthetic tokenism—that hinder its realization. Bridging this divide requires not only design innovation but also institutional commitment to embedding trauma-informed and therapeutic principles across schools, hospitals, and public spaces.

However, critics argue that implementation must be age- and context-specific, particularly for neurodiverse populations. Shimokura et al. (36) found that younger autistic children required calm rooms and outdoor play opportunities, whereas older children benefitted from mainstream-like environments supplemented with sensory-friendly spaces. Similarly, Gawlak & Banaś (38) highlighted the importance of participatory design, calming aesthetics, and user-friendly layouts in youth mental health centers, suggesting that uniform design solutions are unlikely to meet diverse needs.

**Figure 11:**
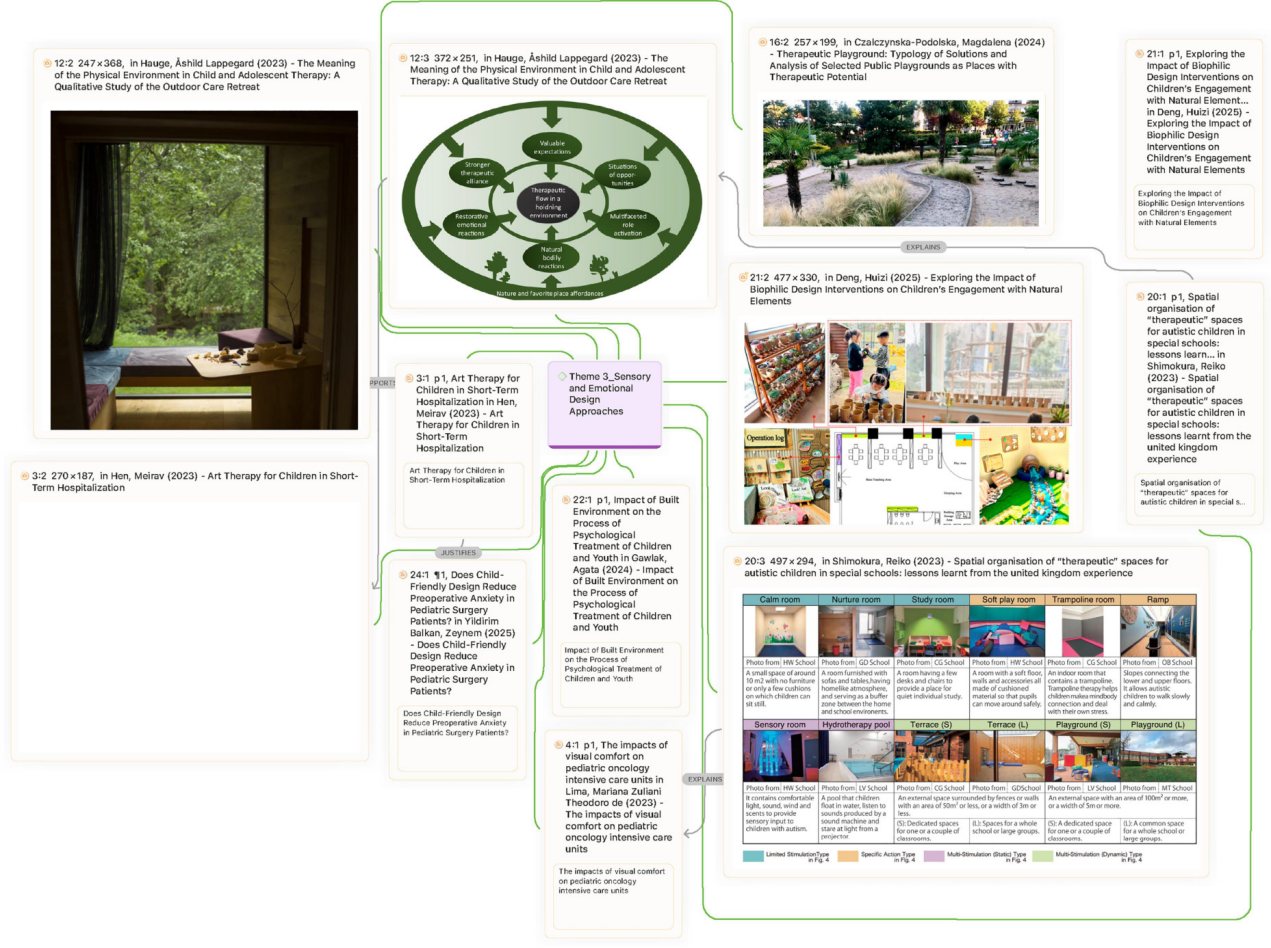
Theme 3 (Sensory and Emotional Design Approaches).

In sum, proponents argue that sensory and emotional features are powerful non-pharmacological interventions that can significantly improve paediatric wellness. Critics counter that without nuanced, context-driven implementation, these features risk being underutilized or reduced to decorative elements. The ongoing challenge, therefore, lies not in affirming their importance, which is well established, but in ensuring that sensory and emotional design is systematically and equitably embedded into paediatric healthcare and educational environments (Figure 11).

#### 3.2.4 Theme 4: inclusive and family-centered design

This theme emphasizes designing with families and prioritizing inclusivity across diverse contexts. Stakeholder participation and family presence consistently emerge as critical determinants of supportive paediatric environments. Babbu & Haqu (34), for example, identified four universal factors shaping healthcare settings—child-centered design, indoor environmental quality, positive distractions, and autonomy/control—all of which were strongly associated with improved outcomes for both children and their families. While not exclusive to paediatrics, these findings suggest that inclusivity is not optional but fundamental to environments that sustain health and wellbeing. In educational contexts, Khan et al. (16) provided empirical evidence for the value of participatory design, showing how co-design processes in schools fostered inclusive outdoor learning environments. Extending the principle into domestic settings, Yoo et al. (24) demonstrated how behaviour-guided smart home environments can support children's autonomy and routines, illustrating that inclusivity must cut across hospitals, schools, and homes alike.

**Figure 12:**
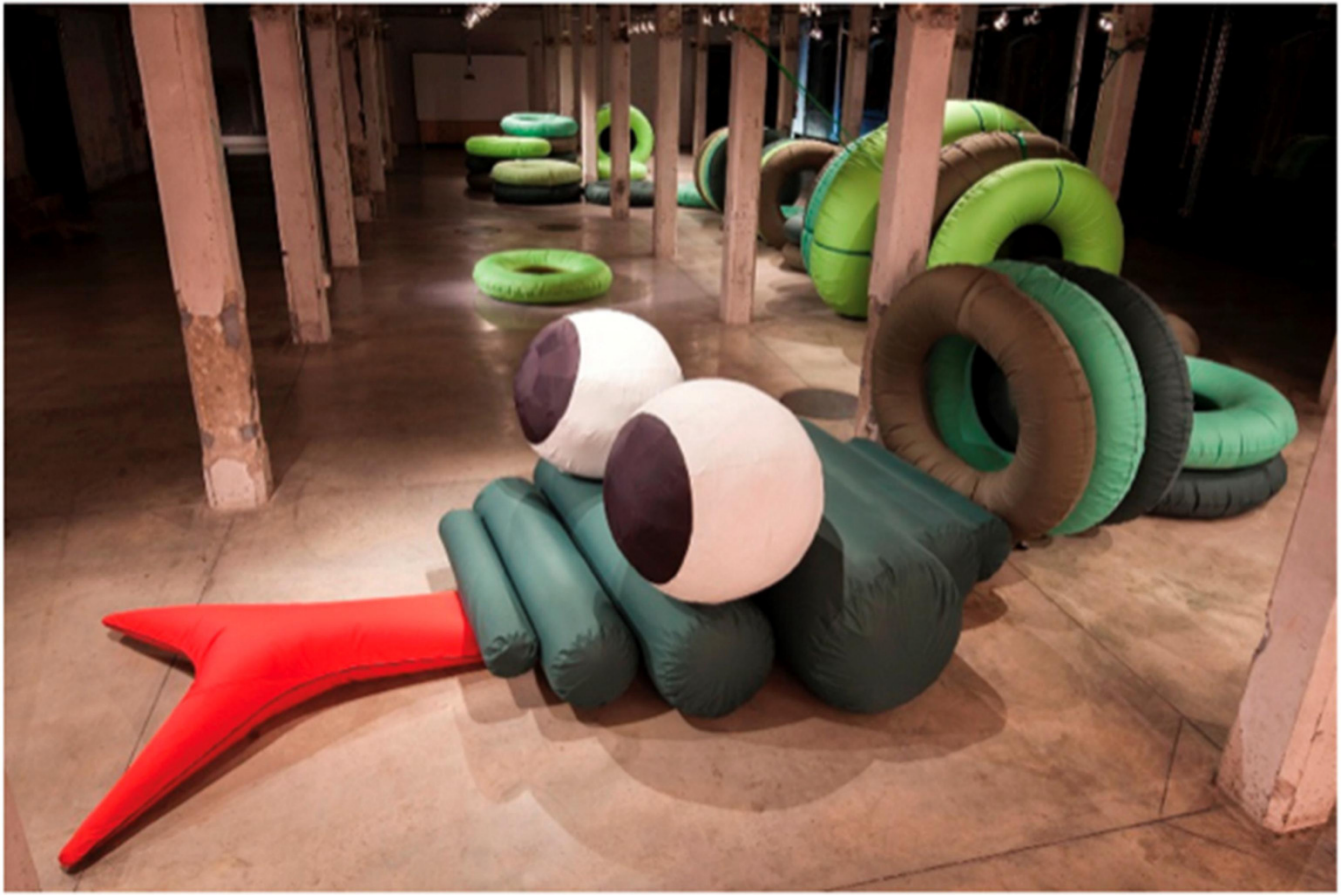
Transformation of artworks into inflatable wheels (35).

At the same time, broader theoretical perspectives complicate this narrative. Szustakiewicz (35), in her analysis of Iza Rutkowska's work, argued that inclusivity cannot be reduced to functional design principles alone. Instead, the creation of “therapeutic spaces” relies on the synergistic interplay of users, spatial conditions, and creative practices that imbue environments with new meanings (Figure 12). Her findings suggest that inclusive design not only benefits individuals directly but also extends to communities, strengthening social relationships and revitalizing alien spaces such as former industrial buildings. This perspective positions inclusive design as both a personal and collective good, challenging narrower interpretations that limit inclusivity to accessibility or comfort.

More specialized evidence highlights the need for carefully differentiated therapeutic spaces, particularly for children with autism spectrum disorder (ASD). Shimokura et al. (36) identified ten distinct types of therapeutic spaces across six UK special schools, organized along two axes—multi-versus limited stimulation and static vs. dynamic. These ranged from calm rooms and nurture rooms to sensory rooms, hydrotherapy pools, terraces, and playgrounds. Her spatial analysis further emphasized the importance of adjacency, noting that classrooms in nurseries or primary schools should ideally connect directly to outdoor environments, while soft play and sensory rooms require proximity to core learning areas. Such findings underscore that inclusivity demands nuanced spatial organization tailored to developmental and neurodiverse needs rather than uniform solutions.

**Figure 13:**
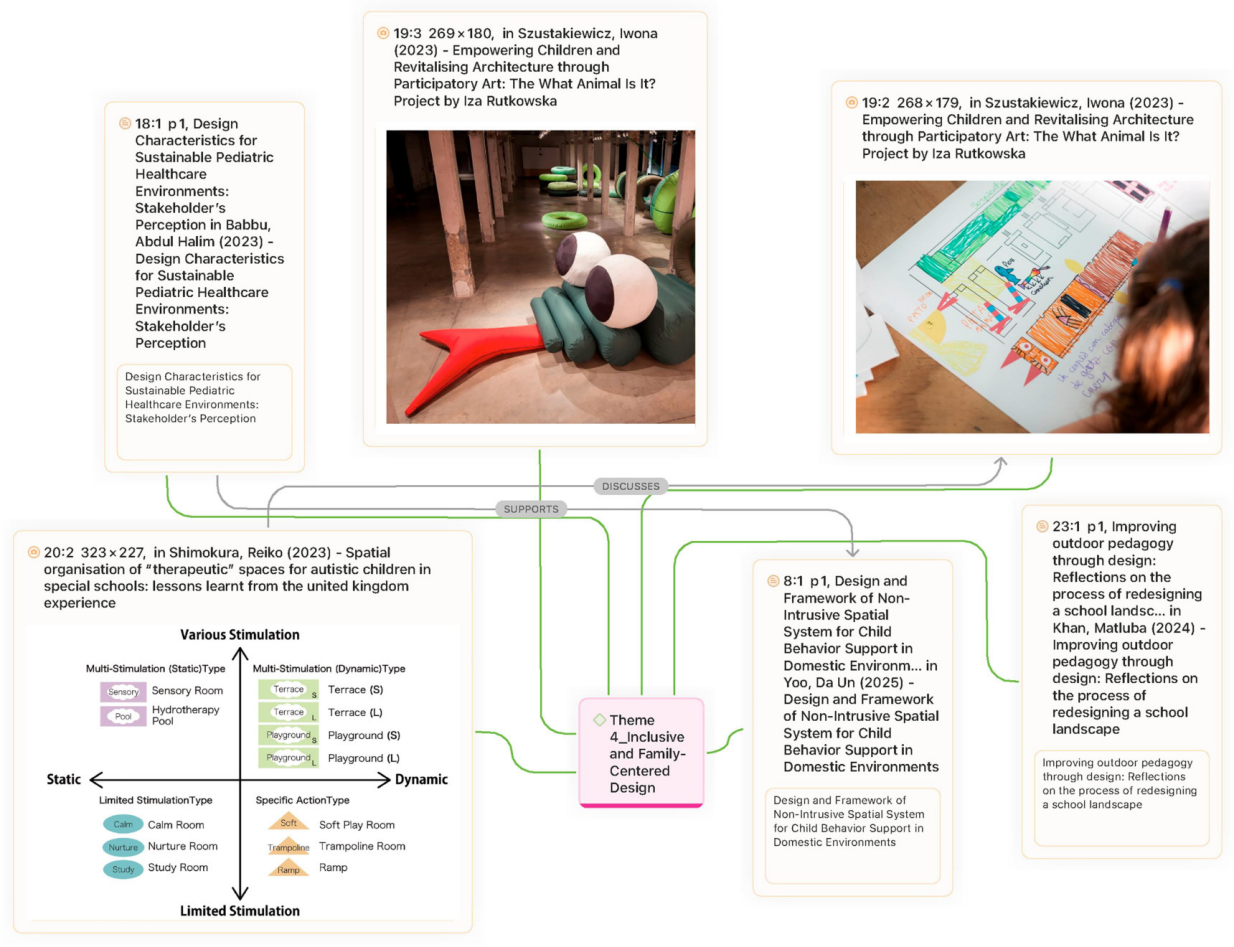
Theme 4 (inclusive and family-centered design).

The central debate, therefore, is not whether inclusivity and family-centered design are necessary; empirical and theoretical evidence clearly affirms their importance, but rather how they are to be achieved in practice. Proponents argue that participatory processes, smart technologies, and differentiated therapeutic spaces provide concrete strategies for embedding inclusivity. Critics, however, caution that design risks becoming tokenistic if it fails to capture the lived practices, creative activities, and contextual differences that transform spaces into genuinely therapeutic environments. Bridging this divide requires an integrated approach that combines universal design principles with sensitivity to cultural, developmental, and contextual variations (Figure 13).

#### 3.2.5 Theme 5: aesthetic, cultural, and identity-oriented design

**Figure 14:**
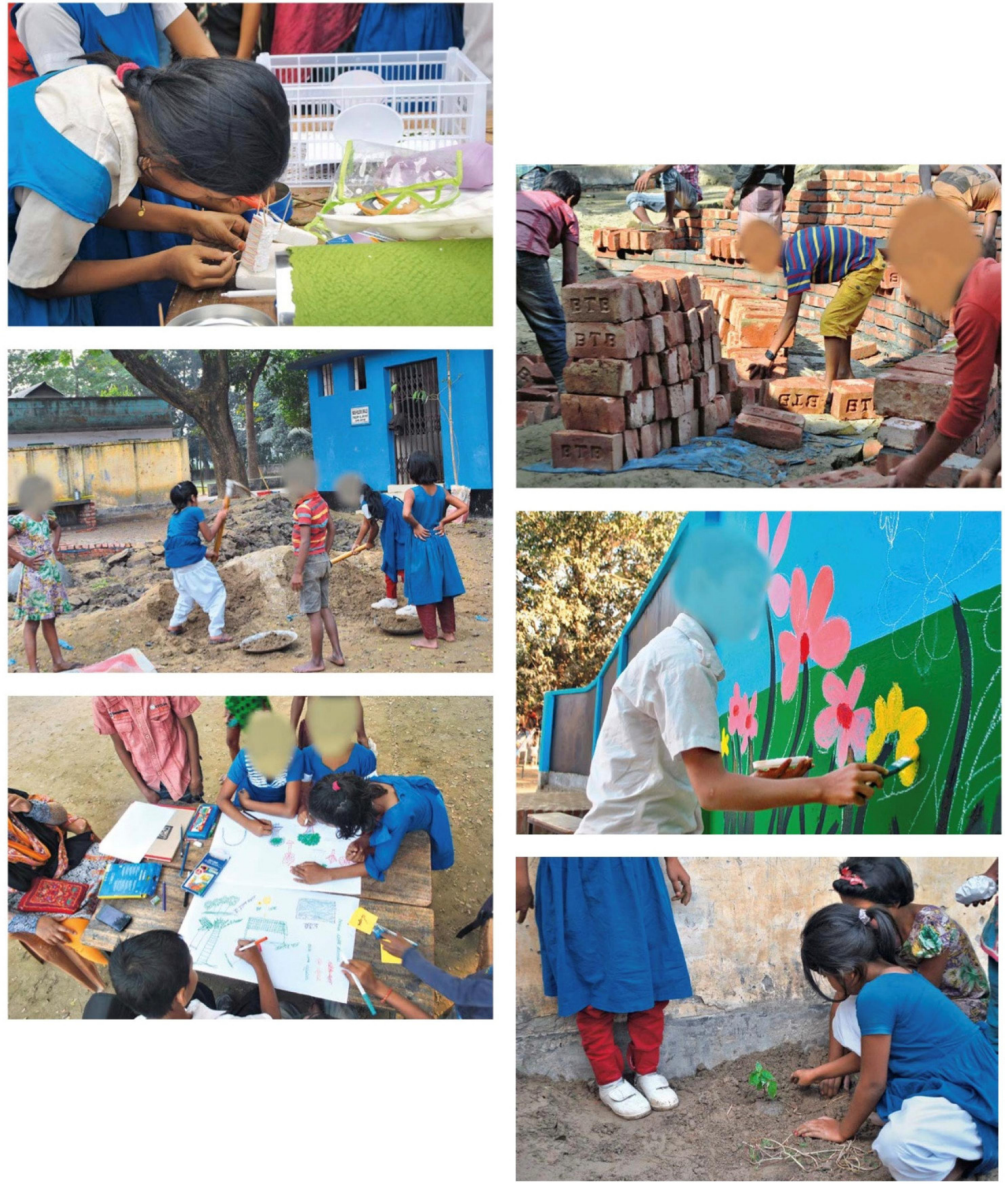
Children actively participated in the design and construction (16).

This theme explores how art, culture, and identity enrich paediatric environments by fostering meaning, empowerment, and belonging. A consistent argument across the literature is that children's well-being is not purely clinical, but also cultural and identity-based. For example, Szustakiewicz (35) examined the What Animal Is It? participatory art project in Madrid, where children co-created playground art in a repurposed warehouse. The process revitalized a neglected site while empowering children as co-authors of space. Similarly, Burke et al. (26) described Art-Well-Being, a museum-based intergenerational program in Melbourne that fostered cultural continuity and resilience through collaborative art-making. In hospital contexts, Hen (41); Yildirim Balkan et al. (39) demonstrated that art therapy and art-infused interiors reduced stress and helped children reclaim identity during vulnerable periods. At the school level, Khan et al. (16) showed how redesigning a Bangladeshi schoolyard with local motifs and native vegetation reinforced cultural identity alongside play and learning (Figure 14). Taken together, these studies highlight the role of participatory and culturally embedded design in cultivating resilience and strengthening ties to community and place.

Yet, critics highlight that cultural enrichment cannot be separated from broader social determinants of health. Wilson et al. (27), in a decade-long housing condition survey of 6,589 cases in Kansas City, demonstrated that external housing conditions, when combined with housing age, were more effective in predicting lead exposure risk than age alone. His findings suggest that cultural and aesthetic interventions, while important, must be accompanied by attention to basic housing quality, which remains a critical determinant of children's health. Similarly (17), synthesized mixed but generally positive findings on the relationship between green space access, physical activity, and childhood obesity outcomes. However, the review also emphasized methodological shortcomings in how exposure to green space is measured, cautioning against simplistic assumptions that cultural or environmental enrichment alone guarantees improved health. These broader structural studies suggest that while participatory art and culturally embedded design empower children, they cannot fully compensate for risks embedded in housing conditions, environmental exposures, and uneven access to urban green space.

**Figure 15:**
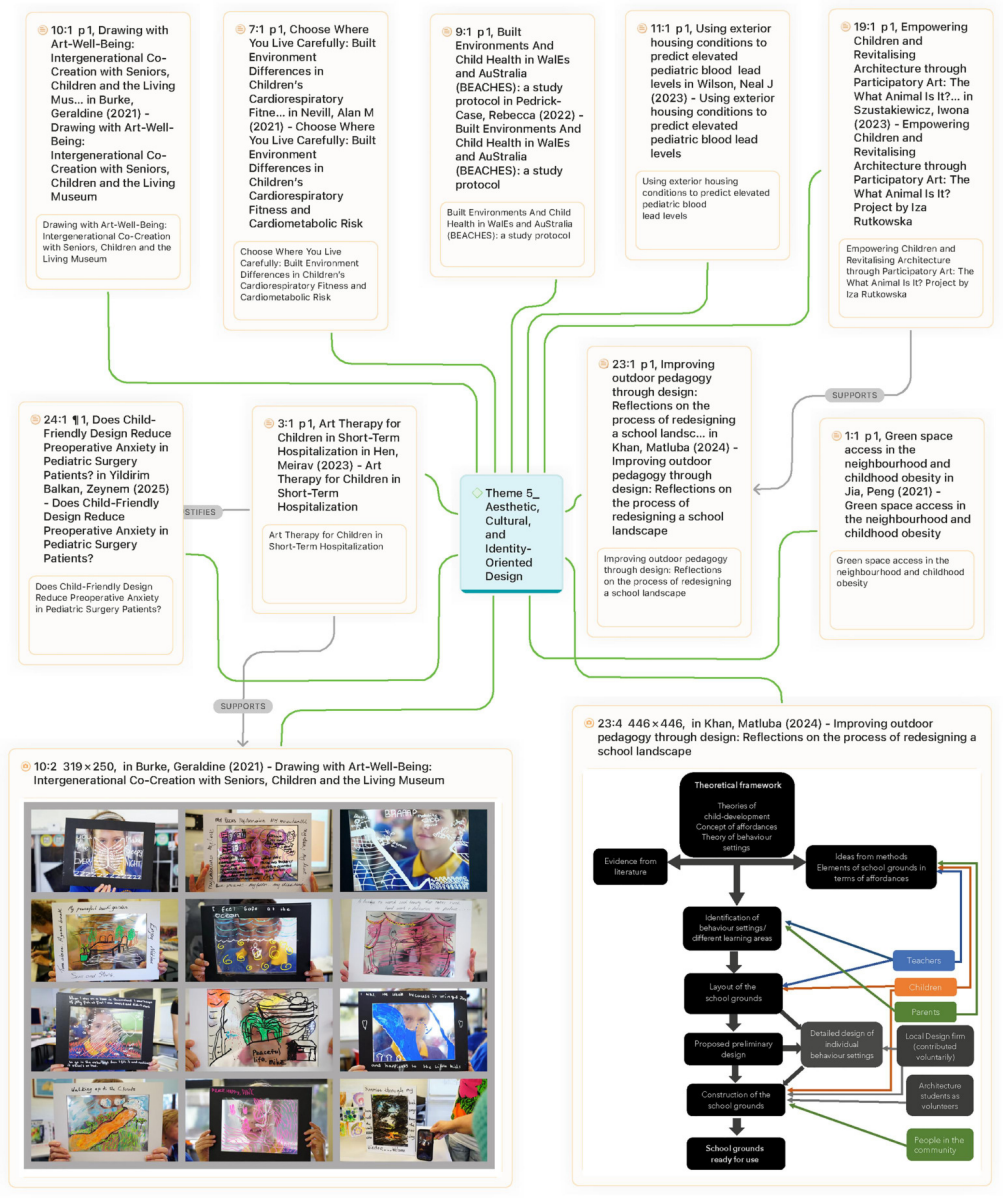
Theme 5 (Aesthetic, Cultural, and Identity-Oriented Design).

The debate, therefore, centers on the balance between enrichment and equity. Proponents argue that embedding art, culture, and identity into pediatric spaces builds resilience, belonging, and empowerment. Critics contend that unless such efforts are integrated with systemic improvements to housing, urban environments, and public health policy, they risk remaining symbolic rather than transformative. Bridging these perspectives requires a multi-scalar approach—one that acknowledges wellness as simultaneously cultural, environmental, and structural, and that situates art and identity not as decorative additions, but as integral to equitable child health and development (Figure 15).

## 4 Discussion and future studies

Future research at the intersection of art, design, paediatric wellness, and the built environment should move toward holistic, cross-thematic integration, examining how therapeutic aesthetics, sustainability, and accessibility interact to influence health outcomes. More rigorous empirical validation is needed through randomized or longitudinal studies assessing interventions such as colour therapy, acoustic treatment, and biophilic elements with both quantitative and qualitative indicators. Research must also broaden its scope to include low-resource and marginalized contexts, where design may have amplified impacts. Greater use of participatory methods involving children and caregivers is essential to capture lived preferences and emotional responses. With the rise of sensor-based and AI-driven environments, investigations should explore the potential and ethical implications of smart technologies in supporting wellness. Finally, future studies should bridge research and practice by developing evidence-based guidelines, post-occupancy evaluation tools, and longitudinal assessments to understand how children experience built environments over time, thereby ensuring that design strategies are sustainable, equitable, and transformative.

## 5 Conclusion

This review asked: *What are the current trends and patterns in art and design for the wellness of pediatrics in the built environment from 2020 to 2025?* The evidence reveals five interrelated themes that define the field. Biophilic and nature-based design highlights the therapeutic role of daylight, greenery, and outdoor access. Playful and interactive environments demonstrate the developmental and social value of play, with therapeutic playgrounds, participatory schoolyards, and smart technologies extending interactivity across settings. Sensory and emotional design emphasizes trauma-informed and neurodiversity-sensitive strategies, where art, light, acoustics, and adaptable layouts function as non-pharmacological supports for stress reduction and emotional regulation. Inclusive and family-centered design positions families and stakeholders as co-creators of supportive environments, ensuring that children's needs are met across healthcare, education, and domestic contexts. Finally, aesthetic, cultural, and identity-oriented design illustrates how participatory art and culturally embedded spaces foster belonging, empowerment, and resilience.

Together, these themes indicate that the dominant trend from 2020 to 2025 is a move toward holistic, integrative frameworks in paediatric environments. Rather than focusing solely on safety or function, contemporary design emphasizes the interplay of nature, play, sensory comfort, inclusivity, and cultural identity. The overall pattern suggests that paediatric wellness is understood as multidimensional—clinical, emotional, social, and cultural—requiring environments that both heal and empower. The challenge for future research and practice lies in translating these advances into scalable, policy-supported solutions that ensure equitable access for all children.

## 6 Implications for policy and practice

The findings of this review reveal critical intersections between art, design, and paediatric wellness that are often underexplored in built environment policies. Decision-makers in healthcare infrastructure development, particularly in paediatric settings, should consider the integration of sensory-friendly designs, child-centered aesthetics, and restorative environmental features as part of standard planning processes. Incorporating evidence-based design elements, such as color psychology, natural lighting, interactive artwork, and flexible spatial layouts, can significantly enhance children's emotional, cognitive, and physical recovery in clinical environments.

From a policy perspective, this review underscores the need for updated architectural and healthcare design guidelines that specifically address paediatric needs. Institutions and government bodies responsible for hospital accreditation and facility standards should adopt a more holistic framework that emphasizes environmental wellness as a component of paediatric care. Furthermore, design practitioners and healthcare administrators should be encouraged to collaborate early in the design process to ensure that spaces are not only clinically functional but also developmentally supportive and emotionally responsive to children's unique needs.
